# Acute Epstein-Barr Virus-Related Myopericarditis in an Immunocompetent Young Adult

**DOI:** 10.7759/cureus.95942

**Published:** 2025-11-02

**Authors:** Nicholas Sze, Rachel Cox, Alexander J Kobzik, Andrew J Berglund

**Affiliations:** 1 Department of Internal Medicine, Wright State University Boonshoft School of Medicine, Dayton, USA; 2 Department of Internal Medicine, Wright-Patterson Medical Center, Dayton, USA

**Keywords:** acute myopericarditis, bowel ischemia, epstein-barr virus, id critical care, immunocompetent host, left ventricular cardiac thrombus, sepsis and septic shock

## Abstract

Epstein-Barr virus (EBV) is a highly prevalent infection that typically presents as a self-limiting, mild illness. In rare cases, EBV infection can cause life-threatening complications, including myopericarditis. This case report describes a 20-year-old male who developed EBV-associated myopericarditis leading to left ventricular thrombus. This embolized to the gastrointestinal tract, leading to bowel necrosis, septic shock, and admission to a medical intensive care unit.

EBV-associated myopericarditis is a rare occurrence, especially for immunocompetent young patients. Thrombus formation in this context is even rarer. This case highlights possible cardiovascular complications of active EBV infection. Clinicians should be aware of the wide array of EBV-associated conditions and maintain suspicion of cardiovascular involvement in the hospital. Patients should be treated at facilities equipped to manage potential cardiovascular involvement if it is suspected.

## Introduction

Epstein-Barr virus (EBV) is a highly prevalent infection that typically presents as infectious mononucleosis, with self-limiting symptoms [1] of fever, pharyngitis, lymphadenopathy, and hepatosplenomegaly [2]. It is estimated that more than 90% of the world’s population carries EBV as a latent infection of B lymphocytes [3]. Severe complications and organ-specific involvement are uncommon in immunocompetent individuals. Cardiovascular complications are rare and described primarily in isolated reports, and recognition of rare presentations is crucial, as delayed diagnosis can result in increased morbidity.

Cardiovascular involvement includes coronary artery aneurysm, pericardial effusion, myocarditis, arrhythmias, and heart failure, and is a rare and serious complication of EBV [1]. The proposed pathogenesis of EBV-associated myocarditis involves viral invasion of cardiac tissue, with a robust immune-mediated response from the host. Subsequent viral replication and cytokine production create widespread inflammation and necrosis, leading to tissue damage [4]. Thrombus has been reported with EBV-associated disease, but not myopericarditis specifically [5-7]. We present a young immunocompetent adult presenting with rare cardiovascular manifestations of EBV infection leading to life-threatening illness.

This case was previously presented as a meeting abstract at the 2024 Ohio and Air Force Chapters Annual Scientific Meeting on October 17, 2024.

## Case presentation

A previously healthy 20-year-old male presented to the emergency department for a two-day history of fever, dizziness, nausea, and non-bloody emesis. He had no history of immunocompromise and had recently returned from a trip to a lake with his friends. He occasionally smoked marijuana and had no known sick contacts.

**Figure 1:**
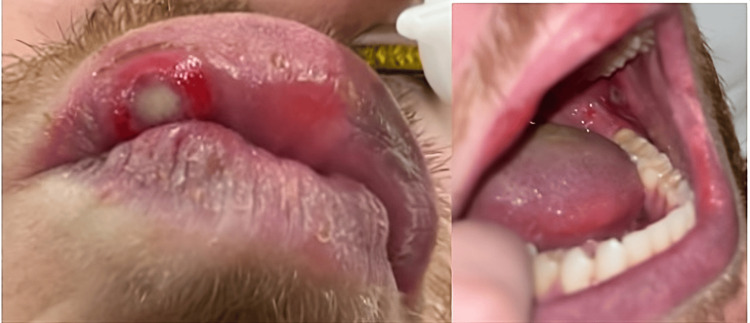
Ulcers of the upper lip and oral mucosa. Shallow lesions with erythematous bases, shallow erosions, and gray centers on the oral mucosa and upper lip.

He was found to have a fever of 103.1°F, tachycardia of 159 beats per minute, respiratory rate of 22, an oxygen saturation of 95% on room air, and hypotension. His blood pressure was 106/47 mmHg after 2 liters of intravenous (IV) fluids were administered. Physical exam revealed multiple painful oral ulcers (Figure 1), a bilateral lower extremity vesicular rash, and diffuse abdominal tenderness.

**Figure 2:**
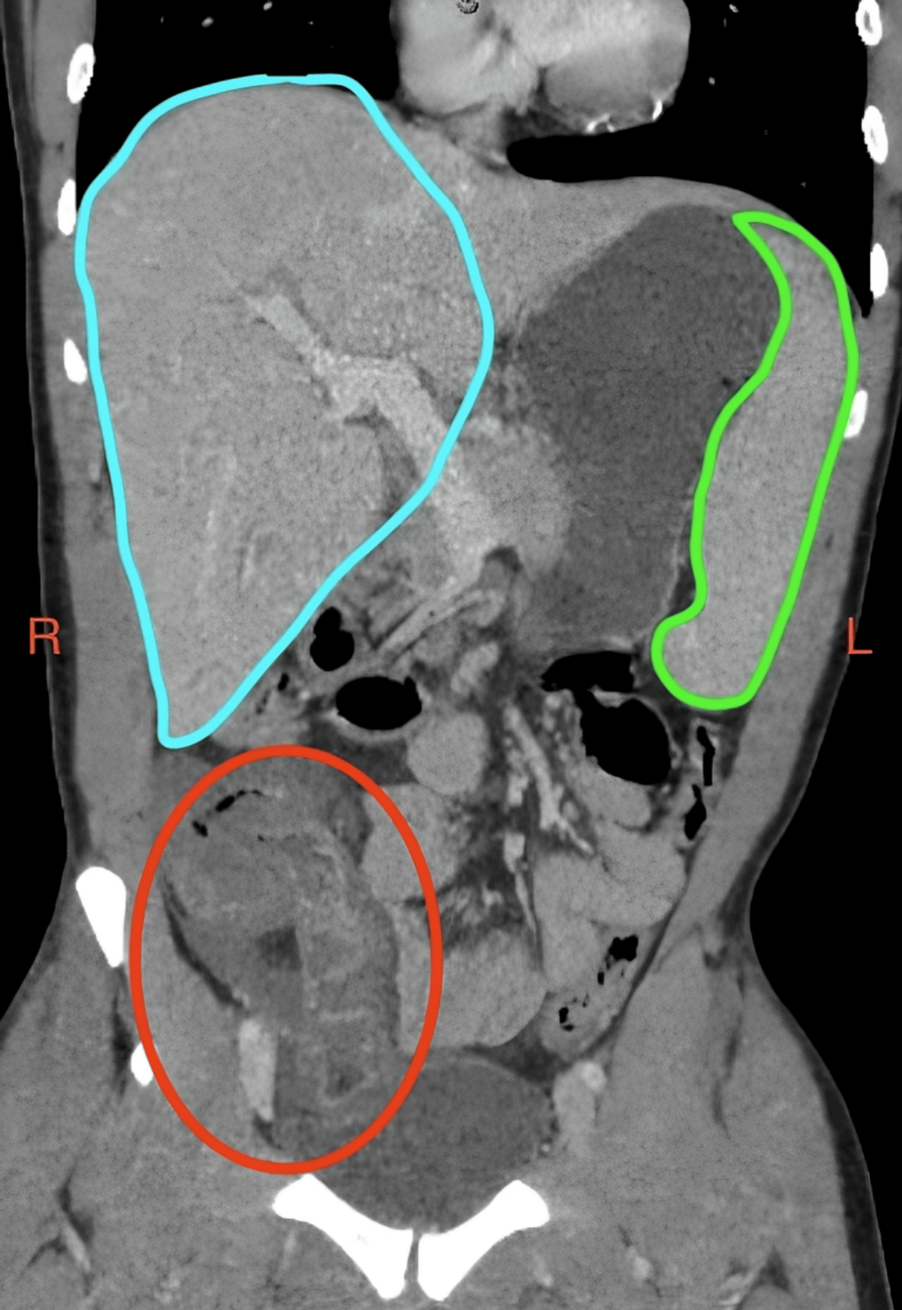
Coronal contrast-enhanced computed tomography of the abdomen and pelvis demonstrating hepatosplenomegaly and ileocolitis. Liver (blue) enlarged to 24 cm in craniocaudal dimension with no intrahepatic or extrahepatic biliary dilation. Spleen (green) enlarged to 17.3 cm in craniocaudal dimension. There is marked thickening of the wall of the terminal ileum, ascending colon, and cecum (red).

Laboratory workup was significant for leukopenia to 3.5 K/μL, elevated creatinine at 1.2 mg/dL, and lactic acidosis of 9.5 mg/dL, among other findings (Table 1). A computed tomography scan of the abdomen revealed hepatosplenomegaly and ileocolitis, leading to intensive care unit (ICU) admission with a diagnosis of septic shock secondary to an intra-abdominal source (Figure 2). He was given a sepsis fluid bolus as previously mentioned, started on broad-spectrum antibiotics, and stress dose steroids.

**Table 1:** Summary of the patient’s initial laboratory findings, including leukopenia and elevated lactic acid, which support a diagnosis of septic shock. WBC: white blood cell count; AST: aspartate aminotransferase; ALT: alanine transaminase; EBV DNA: Epstein-Barr virus deoxyribonucleic acid.

Pertinent labs	Result	Normal range
WBC	3.5 K/μL	4.5-11 K/μL
Lactate	9.5 mmol/L	<3 mmol/L
Creatinine	1.4 mg/dL	Baseline was 0.8 mg/dL
AST	497 U/L	8-33 U/L
ALT	203 U/L	7-56 U/L
Troponin	246 ng/L	<14 ng/L
EBV DNA	Detected	-
Other infectious workup	Negative	-

In the ICU on hospital day zero, the patient was persistently hypotensive despite fluids and increasing vasopressors. Seizure-like activity warranted endotracheal intubation for airway protection. A 24-hour continuous electroencephalogram (EEG) monitoring was initiated, which consisted of a 3-6 Hz delta/theta rhythm and symmetrical normal voltage. There was normal and symmetric sleep architecture, with no electrographic seizures or non-convulsive status epilepticus seen over the entire monitoring period to rule out seizure or epileptic origin. The patient’s lactic acid remained persistently elevated, initially down-trended to 7.8 mg/dL and further to 5.1 mg/dL, but then up-trended to 7.0 mg/dL, prompting concern for an abdominal source of lactic acidosis given his abdominal pain and septic shock. Although he did not have obvious rigidity, he was severely tender to palpation in all four quadrants prior to intubation. Given continuous clinical deterioration, general surgery performed a bedside exploratory laparotomy on hospital day one, which revealed near necrotic-to-necrotic terminal ileum, cecum, and ascending colon, which were resected. An ileostomy was created.

**Figure 3:**
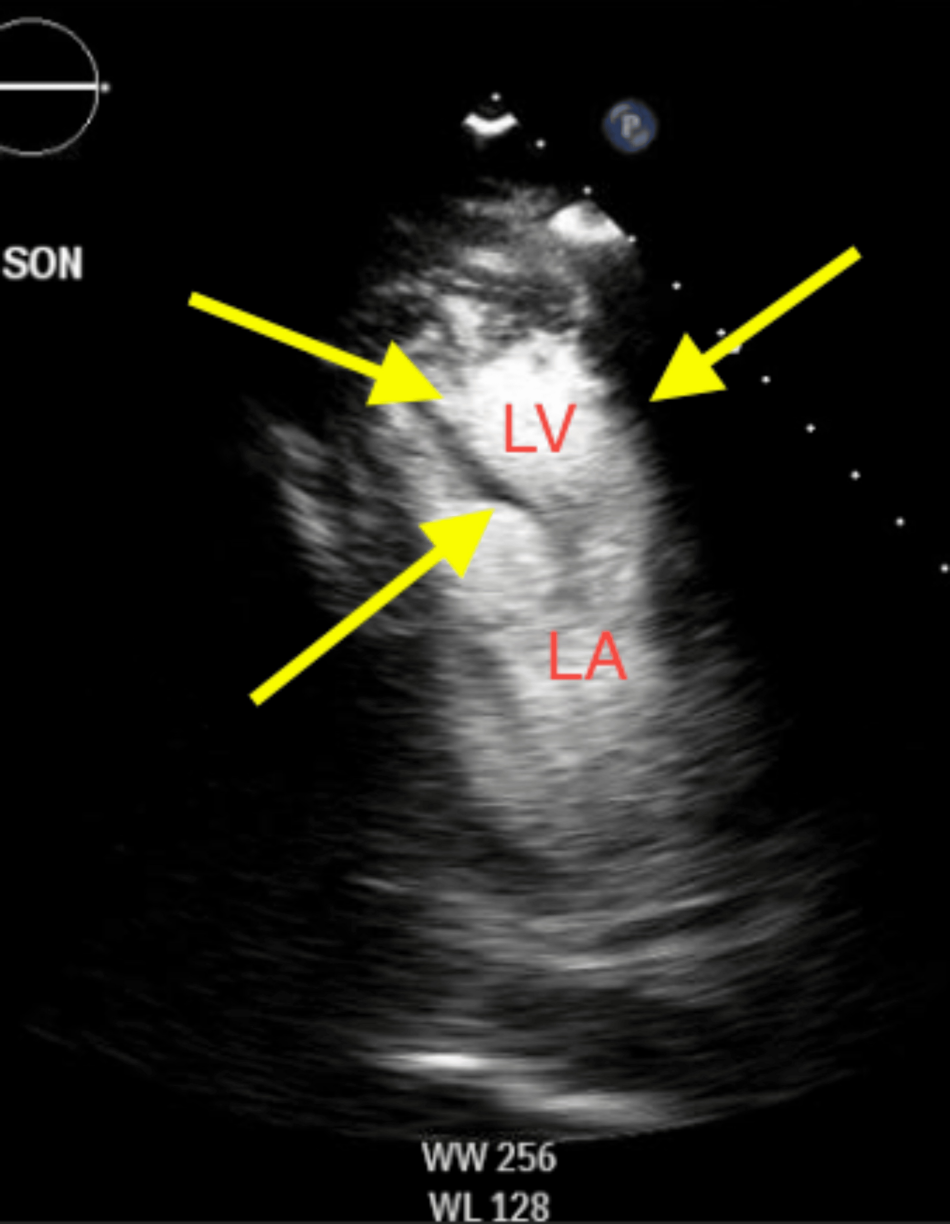
Transthoracic echocardiogram revealing left ventricular apical thrombus (yellow arrows). The two-dimensional apical chamber view depicted thrombus in the left ventricle (yellow arrows). The thrombus is hyperechoic compared to the surrounding myocardial tissue. Right-sided chambers are not well visualized. LV: left ventricle; LA: left atrium.

**Figure 4:**
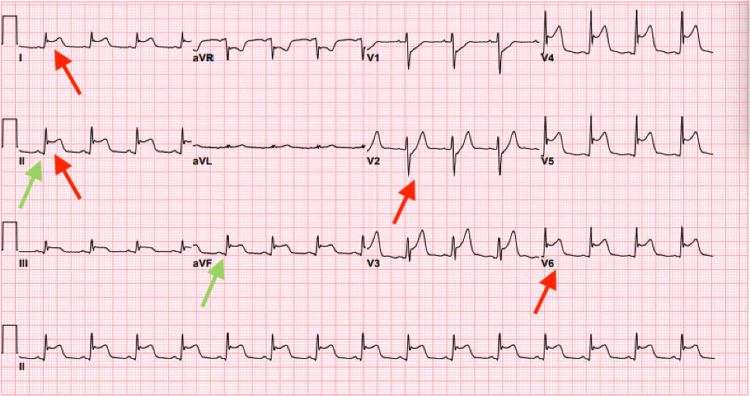
Electrocardiogram showing sinus tachycardia, diffuse ST-segment elevations, and PR-segment depressions, suggesting acute pericarditis. Twelve-lead ECG depicting sinus tachycardia, diffuse ST-segment elevations (shown by red arrows) in leads I, II, and V2-V6, and PR-segment depressions (shown by green arrows) notably in leads II and aVF. This ECG pattern is most consistent with acute pericarditis.

Transthoracic echocardiogram on hospital day two revealed a newly reduced ejection fraction to 20-25% (Figure 3) and left ventricle (LV) apical thrombus with diffuse ST-segment elevation on electrocardiogram (EKG) without pericardial effusion (Figure 4), leading to a diagnosis of myopericarditis. Due to the patient's critically ill state and low clinical suspicion from the consultant cardiologist, no cardiac catheterization was performed. He additionally underwent a lumbar puncture for cerebrospinal fluid (CSF) studies on hospital day two due to seizure-like activity earlier on admission.

Bowel ischemia was determined to be secondary to emboli from LV thrombus, and a heparin drip was initiated. Due to the absence of recent cardiac procedures or other risk factors, an infectious etiology for myopericarditis was suspected. The infectious diseases team was consulted on hospital day zero, prompting a full workup, which was negative except for a positive EBV transplant DNA quantitative panel (Table 2). Due to exposure to fecal contaminated water, primary abdominal infection was considered, but the patient's initial watery diarrhea resolved quickly with supportive management, and further abdominal source workup was not pursued. The hypercoagulability workup for anticardiolipin immunoglobulins, beta-2 glycoprotein, phosphatidylserine immunoglobulins, and anti-prothrombin immunoglobulins was unremarkable. Consistent with his initial presentation, a diagnosis of EBV infection with myopericarditis was made and determined to be the cause of his downstream complications.

**Table 2:** Infectious disease workup. All results listed in the table were negative with the exception of the EBV transplant DNA quantitative panel, which is bolded. RSV: respiratory syncytial virus; STI: sexually transmitted infection; HIV: human immunodeficiency virus; CMV: Cytomegalovirus; PCR: polymerase chain reaction; EBV: Epstein-Barr virus; DNA: deoxyribonucleic acid; VDRL: Venereal Disease Research Laboratory; CSF: cerebrospinal fluid.

Test type	Specific tests/pathogens
Respiratory panel	Rapid COVID, influenza, RSV
Bacterial screens	Group A* Streptococcus* screen, blood cultures, stool pathogen screen, stool *Yersinia*
STI panel	Chlamydia/gonorrhea probe, HIV screen, syphilis screen
Viral PCR/serology	CMV PCR, Coxsackievirus A serology, West Nile virus, meningitis PCR panel, EBV transplant DNA quantitative panel
Fungal antigens	*Histoplasma* (serum/urine), *Blastomyces*, *Aspergillus*
Parasites	*Leptospira* DNA PCR
CSF studies	*Cryptococcus* antigen, VDRL, Gram stain & culture, fungal studies, acid-fast bacilli

**Figure 5:**
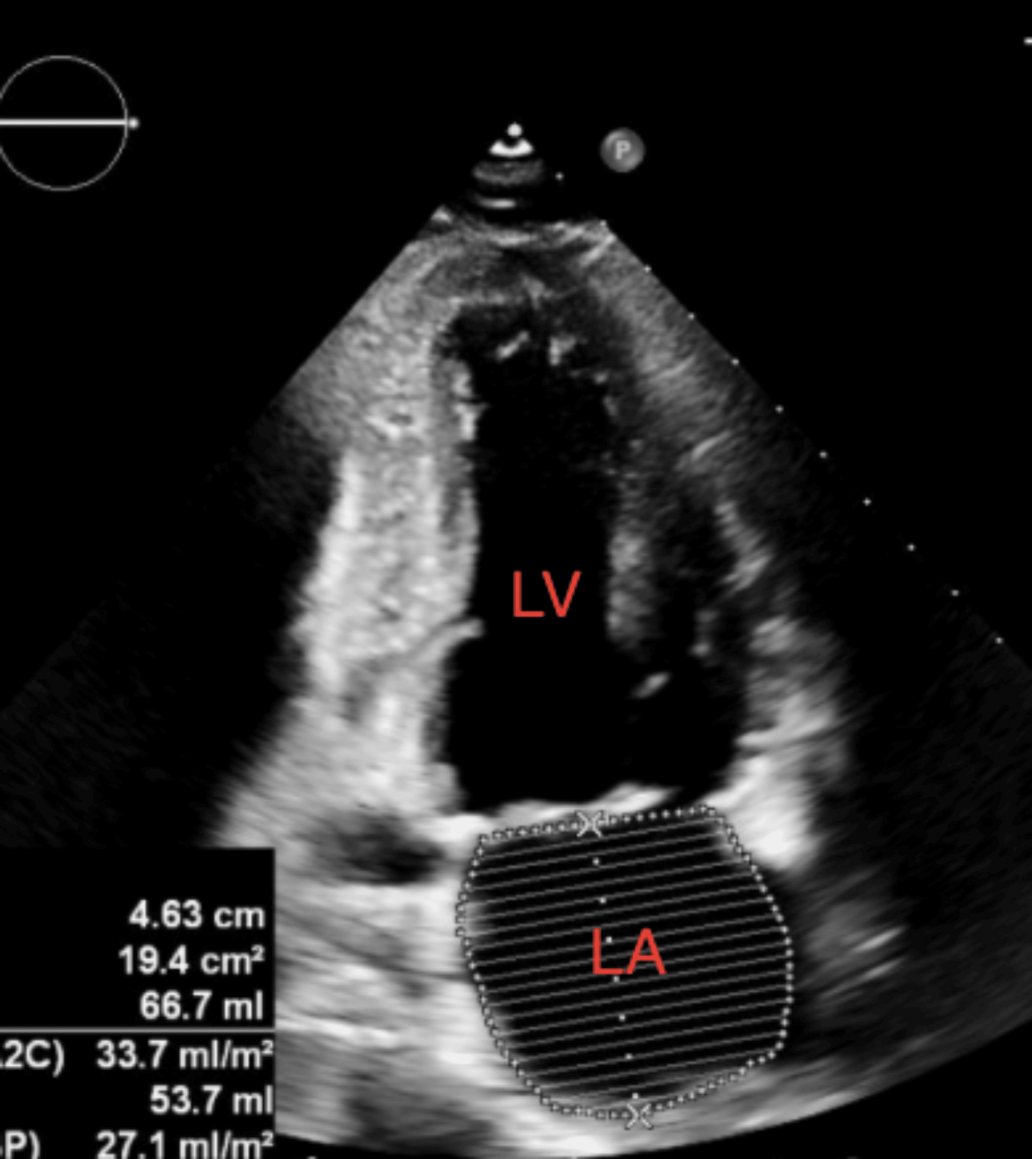
Transthoracic echocardiogram four months after discharge, showing left ventricular ejection fraction of 55%. Two-dimensional apical chamber view. Right-sided chambers are not well visualized. The dotted chamber has no significance. Imaging came from an external facility, and the authors did not have access to the original study. The image above is the only echocardiogram in the chart for this visit. LA: left atrium; LV: left ventricle.

The patient was discharged after a 17-day hospital course. He followed up with primary care, neurology, cardiology, and general surgery in the outpatient setting. A repeat echocardiogram four months after discharge revealed a left ventricular ejection fraction of 55% (Figure 5). He was admitted six months after discharge for elective ileostomy reversal, which was successful, but was re-admitted six days later with abdominal pain and moderate free fluid in the abdomen concerning anastomotic leak. This prompted surgery, revealing bloody ascites of unclear etiology and an intact anastomosis. He had postoperative complications of ileus during this admission. He was subsequently discharged and continues to follow in the outpatient setting with primary care and specialists.

## Discussion

The mechanism behind EBV’s pathogenic effect on the cardiovascular system is not fully understood, but proposed mechanisms suggest lymphocyte clonal expansion, organ invasion, and immune escape may be contributing factors [8]. It is likely that cardiovascular injury is driven by immune-mediated inflammation. EBV induces local tissue inflammation, recruits lymphocytes, increases cell adhesion, and induces cytokine release. The influx of innate immune cells like mast cells, neutrophils, macrophages, and dendritic cells results in an inflammation cascade, ending with remodeling at the site of injury [9]. Cardiovascular involvement in EBV has been documented in several conditions, such as coronary artery aneurysm, myocarditis, various arrhythmias, and heart failure [1].

Although other cases of EBV-associated pericarditis and myopericarditis have been reported [10-13], our case is unique due to the development of left ventricular thrombus with embolization to the bowel in an immunocompetent adult. Thrombus formation due to EBV infection is rare and more commonly associated with CMV. A case report of concomitant infection of EBV and *Cytomegalovirus* (CMV) resulting in portal vein thrombosis has been documented [5]. Interestingly, EBV infection may induce a transient lupus anticoagulant due to seroconversion of viral antibodies, which could lead to thrombus [7]. EBV venous thromboembolism is seldom reported and usually occurs in immunocompromised patients and those with pre-existing thrombus risk [6]. Additionally, there may be an increased risk in young adults [5]. This case represents a rare instance in which EBV-associated myopericarditis resulted in LV bowel thrombus, embolism in the arterial circulation, and severe illness of an immunocompetent adult.

Bowel ischemia from the resultant thrombus likely stemmed from embolization to a watershed area. Regions such as the splenic flexure, the ileocecal region, and the rectosigmoid junction are at high risk due to poor collateral circulation [14].

The gold standard for diagnosing EBV myopericarditis is endomyocardial biopsy with EBV polymerase chain reaction (PCR) [15]. In our case, the EBV transplant quantitative panel was positive, and while it is unclear why other EBV serologies were negative, this case suggests the use of EBV PCR for detecting EBV-associated myocarditis in a less invasive manner. Additional workups include cardiac biomarkers, EKG, echocardiogram, cardiac magnetic resonance imaging (MRI), and coronary angiogram [1].

Management focuses on controlling the immune response caused by EBV. Medications include antiviral therapy (acyclovir and ganciclovir) and anti-inflammatory therapy (glucocorticoids and immunosuppressants). Management of long-term cardiac symptoms should be performed, such as long-term anticoagulation for the risk of future thrombotic events [1]. Our patient did not receive antiviral therapy due to low detected replication, but received glucocorticoids for the presence of septic shock.

## Conclusions

This case highlights the severe, life-threatening complications that can result from EBV infection, even in immunocompetent adults. The consequences of EBV infection, including its risk and pathogenesis of thrombus, are complex and still under investigation. Clinicians should remain vigilant about the potential for cardiovascular involvement, particularly when determining the appropriate level of care and ensuring the treatment center's capabilities align with the patient's needs. Given the potential severity of this condition, patients should be treated at facilities equipped with multidisciplinary teams capable of supporting this advanced disease state. Early recognition and intervention could improve patient outcomes and reduce risk for long-term complications.
